# Novel encephalomyelitis-associated astrovirus in a muskox (*Ovibos moschatus*): a surprise from the archives

**DOI:** 10.1186/s13028-019-0466-0

**Published:** 2019-06-24

**Authors:** Céline Louise Boujon, Michel Christoph Koch, Ronja Véronique Kauer, Elsbeth Keller-Gautschi, Melanie Michaela Hierweger, Stefan Hoby, Torsten Seuberlich

**Affiliations:** 1 NeuroCenter, Division of Neurological Sciences, Vetsuisse Faculty, University of Bern, Bremgartenstrasse 109A, 3012 Bern, Switzerland; 2 Graduate School for Cellular and Biomedical Sciences, University of Bern, Freiestrasse 1, 3012 Bern, Switzerland; 3 Bern Animal Park, Tierparkweg 1, 3005 Bern, Switzerland

**Keywords:** Astrovirus, Encephalitis, Formalin-fixed and paraffin embedded (FFPE), Muskox (*Ovibos moschatus*), Next-generation sequencing (NGS)

## Abstract

**Background:**

The small, single-stranded positive-sense RNA astroviruses are mostly known to be enteric viruses. In recent years, though, different astroviruses were reported in association with neurological disease in various species. In cattle, two distinct neurotropic astrovirus genotype species were described in numerous cases of nonsuppurative encephalomyelitis, with one of these viruses also reported in similar circumstances in several sheep. Here, we retrieved archived formalin-fixed, paraffin-embedded brain tissues of a muskox diagnosed with a comparable disease pattern in 1982 and investigated them for the presence of neurotropic astroviruses with various techniques.

**Results:**

Initially, tissue samples scored positive for both neurotropic astroviruses by immunohistochemistry; however, unexpected results with further immunohistochemical testing, in situ hybridization and qRT-PCR prompted us to submit an RNA extract from the animal’s brain material to next-generation sequencing. We were thus able to obtain the full genome of a novel astrovirus, muskox astrovirus CH18 (MOxAstV-CH18), whose closest relative is an enteric ovine astrovirus. Subsequently, viral RNA could be detected with a specific RT-PCR in the brain of the affected animal, but not in faecal samples from the current muskoxen herd of the animal park where the animal used to be kept.

**Conclusions:**

We identified a novel astrovirus in a historical case of a captive muskox with nonsuppurative encephalomyelitis. Unfortunately, our results and the fact that no material from organs other than of the nervous system was available do not allow any assumption about the epidemiology or pathogenesis of the virus. Still, these findings are yet another piece of evidence that the tropism and species specificity of astroviruses could be more deceptive than generally assumed.

## Background

Muskoxen (*Ovibos moschatus*) are animals native to Arctic regions and belonging to the family *Bovidae*, subfamily *Caprinae*. Although this species is not so common in captivity (115 animals registered in the General ZIMS database as of January 29, 2019 [1]), small herds are kept in some animal parks. Over the last decades, substantial effort was put into the investigation of infectious diseases of free-ranging muskoxen. For instance, parasites infesting these animals were described in numerous studies [2], and several reports about specific outbreaks are available [3–6]. Finally, factors contributing to morbidity and mortality in a declining population of Alaskan muskoxen were investigated in a comprehensive manner [7]. However, the knowledge about neurological diseases of these animals remains limited.

Astroviruses are small, nonenveloped viruses with a genome consisting of single-stranded, positive-sense RNA. The genome includes three overlapping open reading frames (ORFs) flanked by untranslated regions and a poly-A tail, with ORF1a and ORF1b encoding nonstructural proteins (either as nsp1a or nsp1ab through a ribosomal frameshift mechanism) and ORF2 encoding the capsid protein precursor [8]. Within the family *Astroviridae*, members of the genus *Avastrovirus* infect birds, whereas those of the genus *Mamastrovirus* are found in mammals. The taxonomy of astroviruses is currently based on their host species as well as their full capsid protein precursor sequence, with amino acid distances (p-dist) greater than 0.338 defining distinct genotype species [9]. Innumerable strains of these viruses were described from faecal samples of various mammalian species [10]. Apart from humans and minks, in which they are known to cause gastroenteric disease, their association with illness in many animals yet remains unclear. In cattle and sheep, astroviruses were found in diarrheic [11, 12] as well as healthy animals [13, 14]. Besides, astroviruses were reported in association with neurological disease in an increasing number of hosts in recent years: humans [15], minks [16], cattle [17], sheep [18] and pigs [19, 20]. Interestingly, many of these neurotropic astroviruses genetically cluster together in the so-called human-mink-ovine (HMO) clade, of which various enterotropic strains are also part [19].

In cattle, two genotype species were found in cases of nonsuppurative encephalitis: bovine astrovirus CH13/NeuroS1 (BoAstV-CH13/NeuroS1) on the one hand and bovine astrovirus CH15/BH89-14 (BoAstV-CH15/BH89-14) on the other. BoAstV-CH13/NeuroS1 [21] was reported from the USA [17], Switzerland [22], the UK [23], Canada [24, 25] and Japan [26], and could be detected in around one quarter of the cases investigated in retrospective studies [27]. In contrast, BoAstV-CH15/BH89-14 was described in only three cattle up to date [28, 29]. Interestingly, viruses almost identical to this second astrovirus type were reported in several neurologically diseased sheep: they were denominated ovine astrovirus UK/2013/ewe/lib01454 and UK/2014/lamb/lib01455 in two sheep from the UK [18] and ovine astrovirus CH16 (OvAstV-CH16) [30] and CH17 (OvAstV-CH17) [31] in two Swiss cases. Being genetically highly similar to one another, these neurotropic astroviruses of cattle and sheep will be commonly referred to as BoAstV-CH15/OvAstV-CH16.

Since their discovery, we developed several diagnostic tools in order to study these bovine and ovine neutropic astroviruses: in situ hybridization (ISH) [22], immunohistochemistry (IHC) [30, 32] and qRT-PCR [33]. Recently, we were told about a historical case of a captive muskox that was diagnosed with nonsuppurative encephalomyelitis in our division in 1982 (Prof. M. Vandevelde, personal communication), and were therefore curious whether astroviruses also played a role in this case. We retrieved formalin-fixed, paraffin-embedded (FFPE) central nervous system tissue samples of this animal from our archive and investigated these by IHC and ISH for the presence of BoAstV-CH13/NeuroS1 and BoAstV-CH15/OvAstV-CH16. We then extracted RNA from the animal’s brain tissue and performed qRT-PCR as well as next-generation sequencing (NGS) on it. This lead to the discovery of a novel astrovirus.

## Methods

### Tissue samples

FFPE central nervous system tissues (midbrain and thoracic spinal cord) of a muskox (ID 15375) were available from the archive of the Division of Experimental Clinical Research, Vetsuisse Faculty, University of Bern (Bern, Switzerland). The animal was submitted to neuropathological investigation in 1982 after euthanasia because of progressive neurological disease unresponsive to therapy. Original tissues samples had to be re-embedded before further processing. Brain sections of two other muskoxen without neuropathological changes were also available from this archive.

### Faecal samples

Individual faecal samples from all (five) members of the present muskoxen herd of Bern Animal Park were collected between June and July 2018 and stored at 4 °C for a few days, without any additive.

### IHC

Firstly, all brain regions available were screened with our usual IHC protocols for the presence of BoAstV-CH13/NeuroS1 (using hyperimmune serum CH13-ORF2-con) and BoAstV-CH15/OvAstV-CH16 (using hyperimmune serum CH15-ORF2-var). Secondly, samples were tested with other hyperimmune sera: one specific for BoAstV-CH13/NeuroS1 (CH13-23917) and one for BoAstV-CH15/OvAstV-CH16 (CH15-ORF2-var). The generation procedures for hyperimmune sera CH13-ORF2-con, CH15-ORF2-var and CH15-ORF2-con are described elsewhere [30, 32]. Polyclonal antibodies CH13-23917 were obtained by immunizing rabbits with a short polypeptide derived from the capsid protein precursor sequence of our BoAstV-CH13/NeuroS1 index case [22] (ID 45564; amino acids 60–73 of the capsid protein gene of Bovine astrovirus CH13, GenBank accession no. NC_024498.1). The immunization and subsequent affinity purification of the hyperimmune serum were performed at BioGenes GmbH (Berlin, Germany). Regarding the IHC method, tissue sections were first deparaffinised, rehydrated, and endogenous peroxidase activity was blocked in a solution of 3% H_2_O_2_ in methanol. They were then microwave cooked in Dako Target Retrieval Solution, pH 9 (Dako Denmark A/S, Glostrup, Denmark) for antibodies CH13-ORF2-con and CH13-23917, or Dako Target Retrieval Solution, Citrate pH 6 (Dako Denmark A/S, Glostrup, Denmark) for antibodies CH15-ORF2-var and CH15-ORF2-con. Blocking was performed with 10% Goat Serum (Normal) (Dako Denmark A/S, Glostrup, Denmark) in phosphate-buffer saline with 0.5% Tween (PBS-T). The samples were incubated with each primary antibody CH13-ORF2-con (diluted 1:100 in PBS-T), CH13-23917 (diluted 1:50 in PBS-T), CH15-ORF2-var (diluted 1:50 in PBS-T) and CH15-ORF2-con (diluted 1:50 in PBS-T) overnight at 4 °C. Finally, detection was carried out with Dako REAL Detection System (Dako Denmark A/S, Glostrup, Denmark), following the manufacturer’s instructions.

### ISH

The attempt to detect viral RNA in situ was carried out with the RNA Scope Assay [34]. A probe specific for BoAstV-CH15 (RNAScope Probe BoAstV-CH15-C2) was developed and used in combination with a probe specific for BoAstV-CH13/NeuroS1 (RNAScope Probe BovineAstrovirus, already commercially available) in the RNAscope 2.5 HD Duplex Detection Kit (Advanced Cell Diagnostics, Newark, NJ), following the manufacturer’s guidelines.

### RNA extraction

RNA from FFPE material was extracted as described in a study of Delnatte and colleagues [35]. Briefly, two 20 μm-thick sections of FFPE midbrain of muskox 15375 were deparaffinised with xylol and further processed with the RNeasy FFPE kit (Qiagen, Hilden, Germany) according to the manufacturer’s instructions. All assays described below for animal 15375 were performed with the same RNA extract. RNA from faeces was isolated using the QIAamp Viral RNA Mini kit (Qiagen, Hilden, Germany). Faecal samples were first diluted in phosphate buffered saline to a concentration of 20% v/v, centrifuged for 20 min at 4000×*g* and 4 °C, and the supernatant was filtered through a 0.2 μm-filter before being purified according to the manufacturer’s instructions. The positive RNA controls used in this study were extracted with TRI Reagent (Sigma Life Science, St. Louis, MO) from frozen brain tissue of one BoAstV-CH13/NeuroS1-(ID 26875) [33] and one OvAstV-CH16-case (ID 41669) [30]. All RNA extracts were stored at − 80 °C until further processing.

### qRT-PCR for bovine and ovine neurotropic astroviruses

Three or one μL RNA extract from FFPE midbrain tissue of muskox 15375 or frozen brain tissue of animals 26875 and 41669, respectively, were investigated for the presence of astrovirus sequences with the AgPath-ID RT-PCR kit (Ambion, Austin, TX) according to the manufacturer’s instructions. The primer combination CH13-A [33] (targeting ORF1a of BoAstV-CH13/NeuroS1) served for the detection of BoAstV-CH13/NeuroS1, whereas BoAstV-CH15/OvAstV-CH16 was tested with the primer combination CH15 [31] (targeting ORF2 of BoAstV-CH15/OvAstV-CH16). Both assays were run on a 7300 Real Time PCR system (Applied Biosystems, Singapore) with the following conditions: 45 °C for 10 min, 95 °C for 10 min, 40 cycles of 95 °C for 15 s, 62 °C for 20 s, 60 °C for 30 s.

### NGS

Starting material for library preparation was 50 ng RNA extract from FFPE midbrain tissue of muskox 15375. cDNA synthesis was performed without fragmentation with the SMARTer Stranded Total RNA-Seq Kit v2-Pico Input Mammalian (Takara Bio USA, Mountain View, CA), with repeated purifications with AMPure XP beads (Beckman Coulter, Brea, CA). Before single-end sequencing (100 bp) on half a lane with a HiSeq 3000 System (Illumina, San Diego, CA), the library quality was controlled on a Qubit Fluorometer (Life Technologies, Eugene, OR) and a Fragment Analyzer (Advanced Analytical Technologies, Ankeny, IA) with the High Sensitivity NGS Fragment Analysis Kit (Advanced Analytical Technologies, Ankeny, IA).

### De novo assembly

Raw reads were quality-trimmed using trimmomatic (Ver. 0.36). As no reference genome is available for muskoxen, quality-trimmed reads were assembled directly using SPAdes (Ver. 3.12.0). The generated contigs were aligned to viral databases with BLASTN (Ver. 2.7.1+, using viral sequences from GenBank and RefSeq downloaded on July 25, 2018) and DIAMOND (Ver. 0.9.18, using viral sequences from UniProt downloaded on June 13, 2018) on nucleotide and amino acid level, respectively.

### Phylogenetic analysis

Phylogenetic analysis was conducted on the amino acid sequence of the capsid protein precursor (680–842 amino acids in length, all complete) of the novel virus and 44 representative members of the family *Astroviridae.* For the phylogenetic analysis of nonstructural polyprotein nsp1ab amino acid sequences (1256–1369 amino acids in length, all complete), 9 representative members of the family *Astroviridae* were used in addition to the novel virus. All sequences were imported into MEGA (Ver. 7.0.26) and aligned using the built-in MUSCLE alignment tool. Maximum-likelihood trees were generated based on a matrix described by Le and Gascuel [36] with 1000 bootstrap replicates.

### RT-PCR for muskox astrovirus

RT-PCR primers were designed with Geneious 10.1.3 [37] based on the novel astrovirus sequence, with forward primer MOxAstV_F: GGCGGGCCATAGGACTATTC and reverse primer MOxAstV_R: CTTTGGGCATGCTGGAGAGA. One or 4 μL RNA from FFPE midbrain of animal 15375 or muskoxen faecal samples, respectively, were tested using the OneTaq One-Step RT-PCR Kit (New England Biolabs, Ipswich, MA) using the alternative protocol described by the manufacturer.

## Results

### Affected animal

**Fig. 1:**
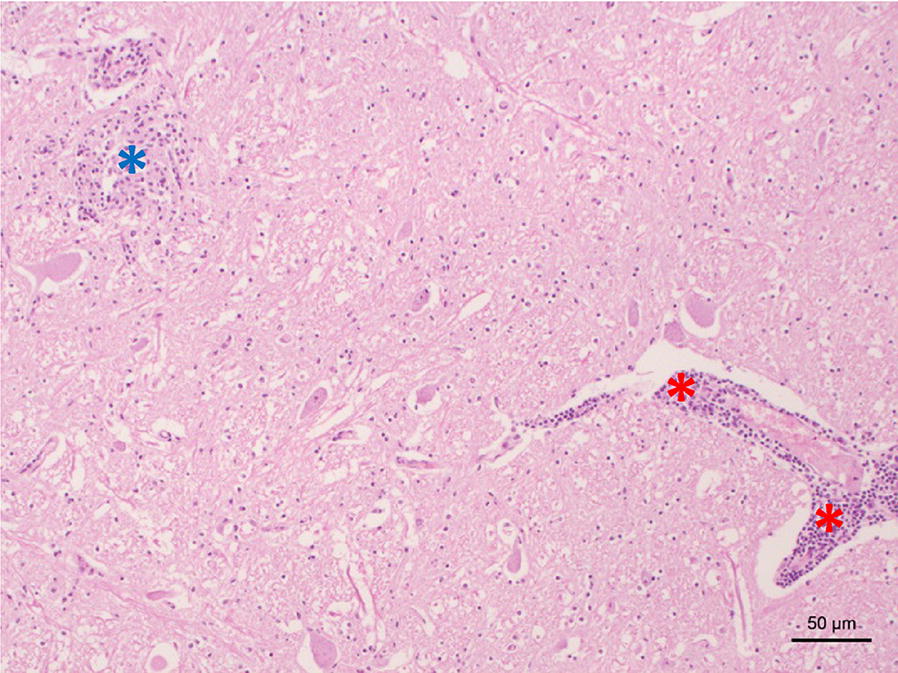
Histopathological changes in the midbrain of muskox (*Ovibos moschatus*) 15375. Note the gliosis on the upper left (blue asterisk) and the perivascular cuff on the lower right (red asterisks). Haematoxylin and eosin stain

In January 1982, a 6-year old male muskox (animal ID 15375) kept at Bern Animal Park (Bern, Switzerland) suddenly showed weakness of the hind limbs, which rapidly progressed to tetraplegia. After 6 days of supportive care without clinical improvement, the animal was released from suffering by a chest hit. Central nervous system tissues were subsequently submitted to diagnostic neuropathological investigation. Histopathologically, all segments of the spinal cord examined as well as the midbrain displayed strong nonsuppurative lesions, particularly in the grey matter, with perivascular cuffs, neuronal degeneration and gliosis (Fig. 1). Although this lesion pattern is indicative of a viral infection, no etiological diagnosis could be pinpointed at that time.

### IHC

**Fig. 2:**
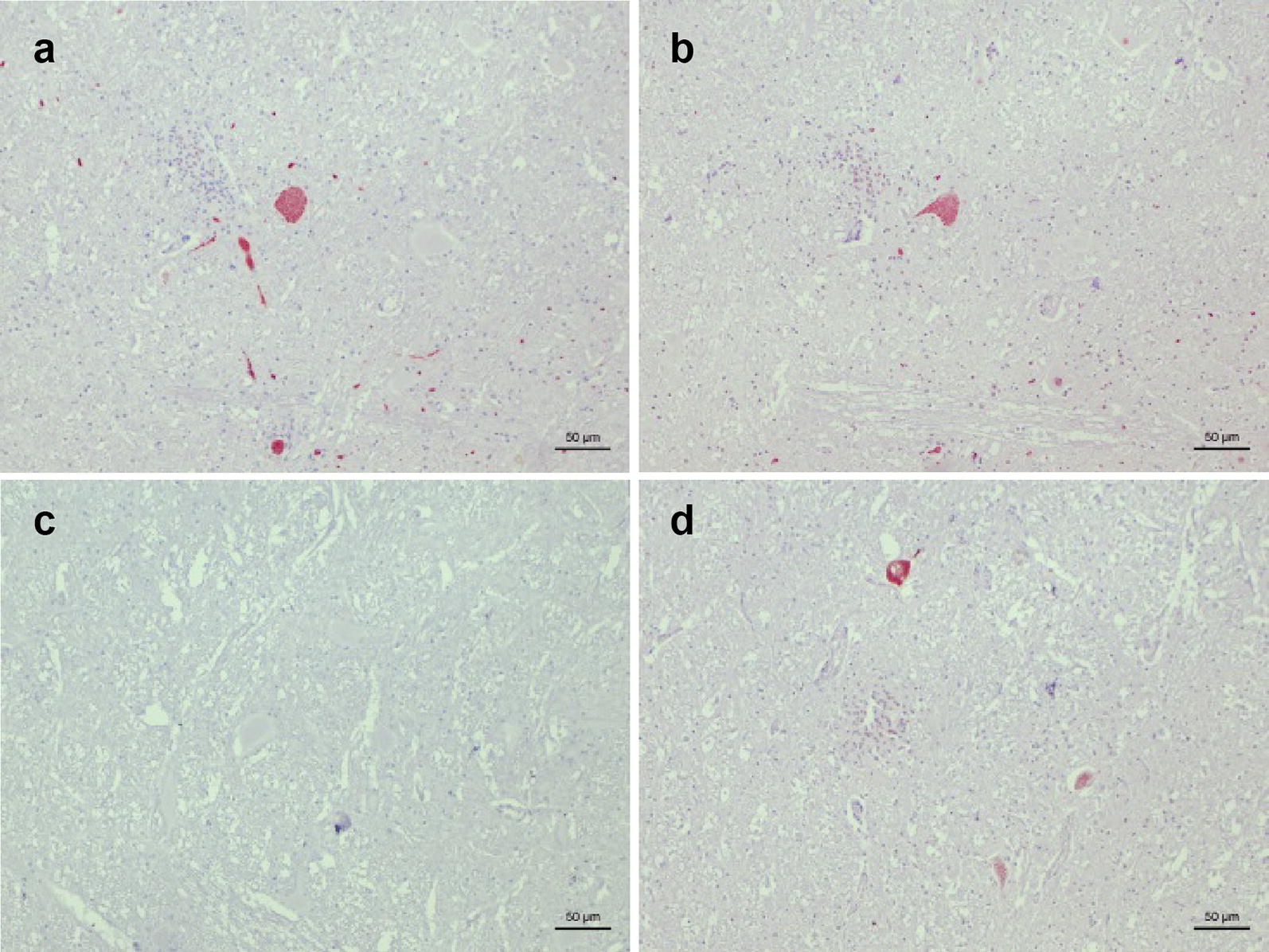
Immunohistochemistry (IHC) for BoAstV-CH13/NeuroS1and BoAstV-CH15/OvAstV-CH16 in the midbrain of muskox (*Ovibos moschatus*) 15375. Red colouring corresponds to positive staining. **a** IHC using hyperimmune antiserum CH13-ORF2-con showing positively stained cells. **b** IHC using hyperimmune antiserum CH15-ORF2-var showing positively stained cells. **c** IHC using hyperimmune antiserum CH13-23917 showing negative staining. **d** IHC using hyperimmune antiserum CH15-ORF2-con showing positively stained cells

Three decades later, we retrieved FFPE central nervous system samples (midbrain and spinal cord) of the animal from our archives. When testing this material for the presence of capsid antigen of BoAstV-CH13/NeuroS1 and BoAstV-CH15/OvAstV-CH16 with a first hyperimmune serum each (CH13-ORF2-con [32] and CH15-ORF2-var [30], respectively), positive staining was obtained for both viruses in all regions investigated (Fig. 2a, b). Subsequently, in an attempt to confirm our findings, we used a second hyperimmune serum for each virus (CH13-23917 and CH15-ORF2-con [30], respectively), and obtained discrepant results: negative staining for BoAstV-CH13/NeuroS1 (conversely, our index case for BoAstV-CH13/NeuroS1, cow 45664 [22], reacted positively), contrasting with a distinctly positive staining for BoAstV-CH15/OvAstV-CH16 (Fig. 2c, d). Brain tissue sections of two other muskoxen without pathological changes were negative with all antibodies.

### ISH

We then tested all available brain regions of muskox 15375 with a dual ISH protocol for the detection of BoAstV-CH13/NeuroS1 and BoAstV-CH15/OvAstV-CH16. Whereas our BoAstV-CH13/NeuroS1 [33] and BoAstV-CH15/OvAstV-CH16 [30] controls both reacted positively for the individual viruses, all muskox samples remained negative.

### qRT-PCR for bovine and ovine neurotropic astroviruses

As the strongest staining in IHC was observed in the midbrain of animal 15375, we extracted RNA from this brain region. We investigated this purified RNA with two qRT-PCR protocols, one specific for BoAstV-CH13/NeuroS1 [33], the other for BoAstV-CH15/OvAstV-CH16; both scored negative.

### NGS and sequence analysis

**Fig. 3:**
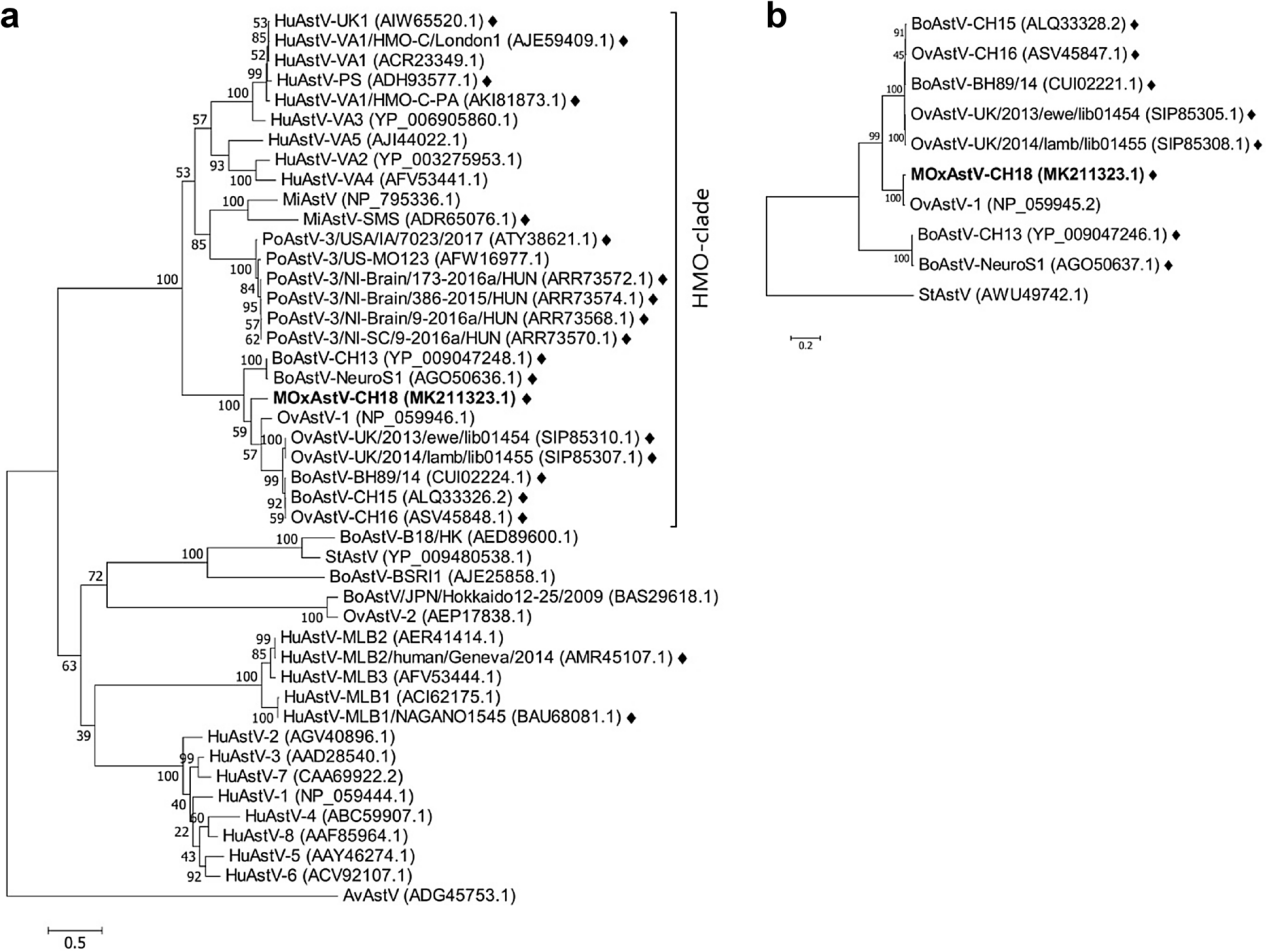
Phylogenetic analysis. Maximum-likelihood trees constructed with **a** the capsid protein precursor (encoded by ORF2) and **b** the nonstructural polyprotein nsp1ab (derived from the common translation of ORF1a and ORF1b) full-length amino acid sequences of the new astrovirus strain MOxAstV-CH18 and selected astroviruses. Both trees demonstrate the close relationship of MOxAstV-CH18 to ovine astrovirus 1 (OvAstV-1). Note that various ovine and bovine neurotropic astroviruses also cluster with MOxAstV-CH18 and OvAstV-1. GenBank accession numbers are shown in brackets. Neurotropic strains are indicated with rhombi. Scale bars illustrate p-distances. AvAstV, avian astrovirus; BoAstV, bovine astrovirus; HuAstV, human astrovirus; MiAstV, mink astrovirus; OvAstV, ovine astrovirus; PoAstV, porcine astrovirus; StAstV, Sichuan takin astrovirus

Despite the inconclusive results obtained by qRT-PCR, we chose to submit the RNA extract from muskox 15375s midbrain to NGS. As RNA from FFPE tissue, especially if old, can be expected to be strongly fragmented, we sequenced 100 bp-long reads in single-end mode, and obtained 198,031,783 of them. After quality-trimming, 186,002,398 reads were used for assembly. Three contiguous sequences (contigs) ≥ 500 nt long and displaying a similarity to astroviruses on nucleotide and/or amino acid level were generated and finally reassembled. The complete sequence obtained (GenBank accession no. MK211323.1) was 6515 nt long, with a series of adenines at the 3′ end corresponding to the virus’s polyadenylated tail. No RACE was carried out to determine the exact ends of the viral genome. The genome contained three putative overlapping ORFs, with a characteristic ribosomal frameshifting signal at the ORF1a/ORF1b junction. The sequence translated from ORF1ab was 1351 and from ORF2 762 amino acids in length, respectively. ORF1ab displayed 87.8% (97.6%, resp.) and ORF2 70.5% (74.6%, resp.) nucleotide (amino acid, resp.) identity to their best hits, which were both on ovine astrovirus 1 (OvAstV-1, that was isolated from the faeces of diarrheic lambs [11, 38]; GenBank accession number NC_002469.1). Phylogenetic analyses based on capsid protein precursor and nonstructural polyprotein amino acid sequences confirmed that the closest relative of the novel astrovirus is OvAstV-1 (Fig. 3). The p-dist between the capsid protein precursor of these viruses is 0.257, which classifies them as the same genotype species (Mamastrovirus 13) according to the present standards of the International Committee on Taxonomy of Viruses [9]. Finally, other bovine and ovine neurotropic astroviruses also clustered in the same branch of the phylogenetic tree.

### RT-PCR for muskox astrovirus

We designed RT-PCR primers based on the sequence of the novel astrovirus obtained by NGS and our bioinformatics pipeline. The RNA extract from FFPE midbrain tissue of muskox 15375 used for NGS produced an amplicon of the expected size (108 bp). Besides, as most mamastroviruses are enteric viruses, we wondered whether the novel astrovirus is to be commonly found in muskoxen’s faeces. However, RNA extracted from faecal samples of the five current muskoxen herd members of the zoo where muskox 15375 was kept 30 years ago remained negative for the virus.

## Discussion

We report a novel astrovirus, discovered in association with a case of nonsuppurative encephalomyelitis in a captive muskox that was euthanized in 1982 because of neurological signs. After initial immunohistochemical reactivity for two neurotropic astroviruses previously reported in cattle and sheep, contradictory outcomes of additional investigations prompted us to submit an RNA extract from FFPE brain tissue of the animal to NGS. We thus obtained the full-length sequence of an astrovirus, which we tentatively name muskox astrovirus CH18 (MOxAstV-CH18), and whose closest relative is an ovine enteric astrovirus, OvAstV-1 [11, 38]. According to current classification criteria, MOxAstV-CH18 belongs to the same genotype species as OvAstV-1 [9].

Cross-reactivity in our IHC assays could be explained by some degree of antigenic similarity between MOxAstV-CH18 and bovine and ovine neurotropic astroviruses. Indeed, numerous stretches up to 37 amino acids in length are conserved among the capsid protein precursors of these viruses. For three of the polyclonal antisera we used in IHC, the viral antigens used to obtain them consisted of 313 to 373 amino acids; some of their epitopes are thus probably also present in the capsid protein of MOx-AstV-CH18. Conversely, the amino acid sequence corresponding to a 16 amino acid-long peptide used to obtain some BoAstV-CH13/NeuroS1-specific antibodies (CH13-23917) that reacted negatively in IHC is not found in MOxAstV-CH18. Similarly, there is probably too much variation at nucleotide level for the dual ISH and both qRT-PCRs specific for BoAstV-CH13/NeuroS1 and BoAstV-CH15/OvAstV-CH16 to recognize MOxAstV-CH18 in brain tissue samples of animal 15375.

As we did not have other muskoxen cases with comparable disease in our archive, we could not investigate further whether MOxAstV-CH18 occurs regularly in such circumstances. Moreover, as we could not find specific reports about neuroinfectious diseases in muskoxen in the literature, astrovirus-associated encephalomyelitis is probably an exceptional finding in this species. Yet, in order to investigate whether MOxAstV-CH18 is a common enteric virus of muskoxen, we tested by RT-PCR several faecal samples obtained from the current herd of the animal park where muskox 15375 was kept, but all were negative. This inconclusive finding therefore leaves open all speculations about the epidemiology and pathogenesis of the virus. Still, the fact that the closest relative of MOxAstV-CH18 is an astrovirus that was isolated from diarrheic lambs [11, 38] raises the question of inter-species transmission. Indeed, even though astroviruses are generally assumed to be host-specific, an increasing number of studies puts this assumption into question [10]. Moreover, our results highlight the potential hazard that the proximity of sheep could represent to the health status of muskoxen populations. Sheep were already considered to be the most probable origin of two epizootics in Norwegian muskoxen: one of contagious ecthyma (orf) [4] and one of pneumonia due to *Mycoplasma ovipneumoniae* [5].

Recently, an astrovirus was described from the faeces of a Sichuan takin (*Budorcas taxicolor* ssp*. tibetana*) [39]. Takins belong to the subfamily *Caprinae*, as muskoxen do. Interestingly, phylogenetic analysis showed that MOxAstV-CH18 genetically cluster together with an ovine faecal astrovirus, in a clade distant from that of the takin astrovirus and bovine enteric counterparts. Differences in tropism might explain the genetic divergence of the viruses.

Because of treatment with formalin, the integrity of nucleic acids extracted from FFPE tissues is generally assumed to be compromised, with fragmentation and cross-linking of molecules [40]. In cancer research, however, FFPE tissue is increasingly considered a valuable source of nucleic acids to study [41]. Conversely, the number of virological studies performed with such material is sparse, with relatively few of them using NGS [42]. In that regard, the most prominent example is probably the determination, in one NGS run, of the full genome of the 1918 pandemic influenza strain that previously took 9 years to complete with traditional sequencing methods [43]. Yet, here we were able to recover the whole genome length of a novel astrovirus from FFPE brain tissue by NGS and de novo assembly. These results therefore demonstrate the power of this approach, also in such conditions, and support its use for viral discovery in archived material as well, highlighting the huge potential for retrospective investigations of unresolved cases or even epidemics.

## Conclusions

Our data indicate that MOxAstV-CH18 is a possible cause of nonsuppurative encephalomyelitis in muskoxen. This warrants further investigation into the spectrum of diseases (in particular of the nervous system) affecting captive and wild muskoxen, as well as other ruminant species. We also show that NGS enables straightforward virus discovery also when applied to FFPE tissues. Finally, the close phylogenetic and antigenic relationships of MOxAstV-CH18 to other ruminant neurotropic astroviruses further question the concept of a strict host specificity for this virus family.

## Data Availability

The raw data generated by next-generation sequencing can be found in the European Nucleotide Archive under Accession Number ERS3126950. The genome of MOxAstV-CH18 is available in GenBank under Accession Number MK211323.1.

## Abbreviations

BoAstV-CH13/NeuroS1: bovine astrovirus CH13/NeuroS1
BoAstV-CH15: bovine astrovirus CH15
FFPE: formalin-fixed, paraffin-embedded
IHC: immunohistochemistry
ISH: in situ hybridization
MOxAstV-CH18: muskox astrovirus CH18
NGS: next-generation sequencing
OvAstV-CH16: ovine astrovirus CH16
OvAstV-CH17: ovine astrovirus CH17
